# Supplementary material for: Efficient Photocatalytic Hydrogen Evolution over TiO2-X Mesoporous Spheres-ZnO Nanorods Heterojunction
Source: Nanomaterials (Basel). 2020 Oct 22;10(11):2096. doi: 10.3390/nano10112096 (PMC7690575; doi:10.3390/nano10112096)
Supplement: Supplementary file 1 [file nanomaterials-10-02096-s001.pdf]

nanomaterials

Supporting Information

Efficient Photocatalytic Hydrogen Evolution over TiO_{2-X} Mesoporous Spheres-ZnO Nanorods Heterojunction

BingKe Zhang [1,2], Qi Li [3], Dongbo Wang [1,2,*], Jinzhong Wang [1,2,*], Baojiang Jiang [3,*], Shujie Jiao [1,2,*], DongHao Liu [1,2], Zhi Zeng [1,2] , ChenChen Zhao [1,2], YaXin Liu [1,2], ZhiKun Xun [4,5,*], Xuan Fang [6,*], ShiYong Gao [1,2], Yong Zhang [1,2] and LianCheng Zhao [1,2]

1 National Key Laboratory for precision Hot Processing of Metals, Harbin Institute of Technology, Harbin 150001, China; zhangbingke007@163.com (B.Z.); 18245019907@163.com (D.L.); 1144420106@hit.edu.cn (Z.Z.); zhaochenstu@163.com (C.Z.); lyx15545582475@163.com (Y.L.); gaoshiyong@hit.edu.cn (S.G.); yongzhang@hit.edu.cn (Y.Z.); lczhao@hit.edu.cn (L.Z.)
2 Department of Optoelectronic Information Science, School of Materials Science and Engineering, Harbin Institute of Technology, Harbin 150001, China
3 School of Chemistry and Materials Science,Heilongjiang University Heilongjiang Univ, Minist Educ Peoples Republ China, Sch Chem & Mat Sci, Key Lab Funct Inorgan Mat Chem, Harbin 150080, China; liqchem@sina.com
4 College of Science, Guangdong University of Petrochemical Technology, Guandu Road No. 139, Maoming 525000, China
5 Key Laboratory for Photonic and Electronic Bandgap Materials, Ministry of Education, School of Physics and Electronic Engineering, Harbin Normal University, Harbin 150025, China
6 State Key Laboratory of High Power Semiconductor Lasers, School of Science, Changchun University of Science and Technology, 7089 Wei-Xing Road, Changchun 130022, China
* Correspondence: wangdongbo@hit.edu.cn (D.W.); jinzhong_wang@hit.edu.cn (J.W.); shujiejiao@hit.edu.cn (B.J.); jbj@hlju.edu.cn (S.J.); xuzhikunnano@163.com (Z.X.); fangx@cust.edu.cn (X.F.)

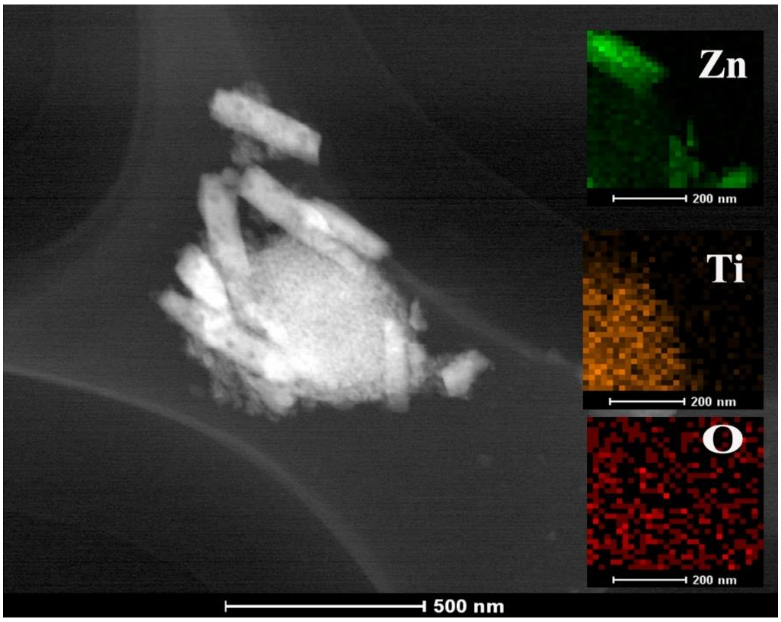

Figure S1. EDS elemental mapping images of the ZnO NRs/TiO_{2-x} MCs.

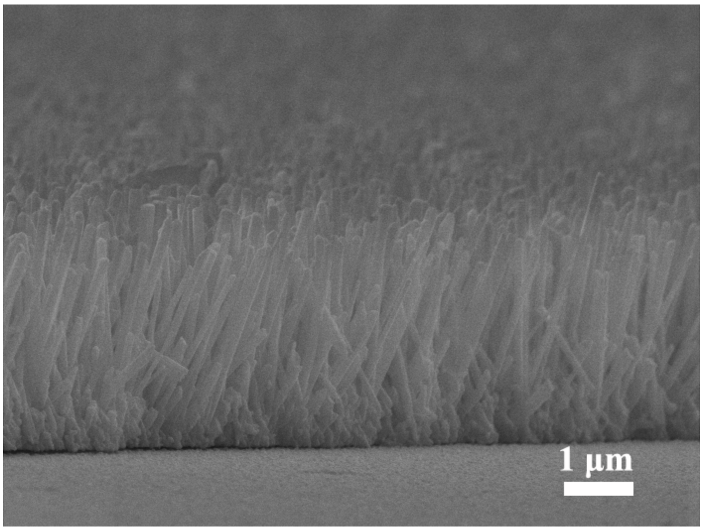

Figure S2. Cross-section of pure ZnO nanorods.

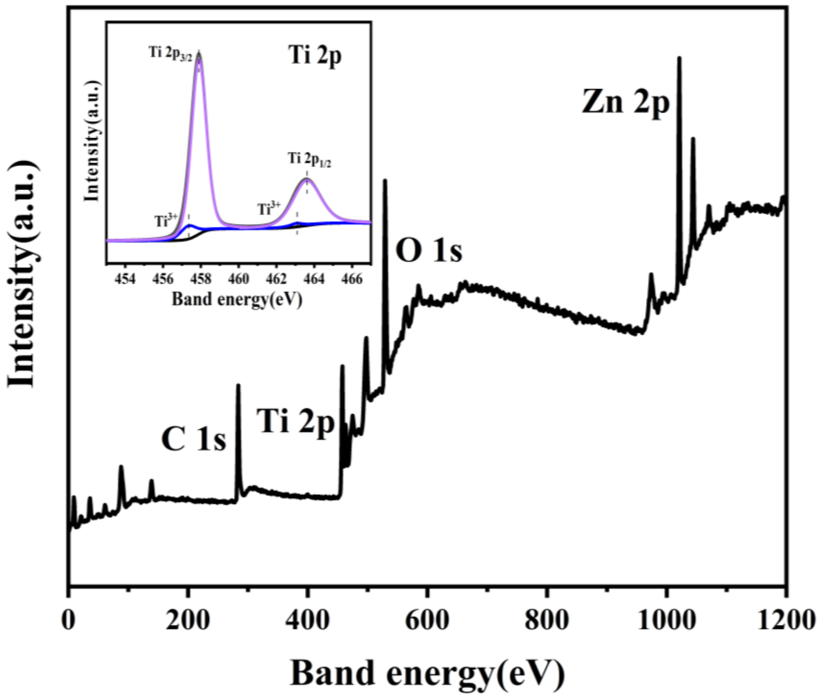

Figure S3. XPS survey spectrum of the ZnO/TiO_{2-X}. Inset: exact XPS analysis of the inner Ti 2p orbital electrons(peak area Ti^{3+}/Ti^{4+} =0.0758).

.

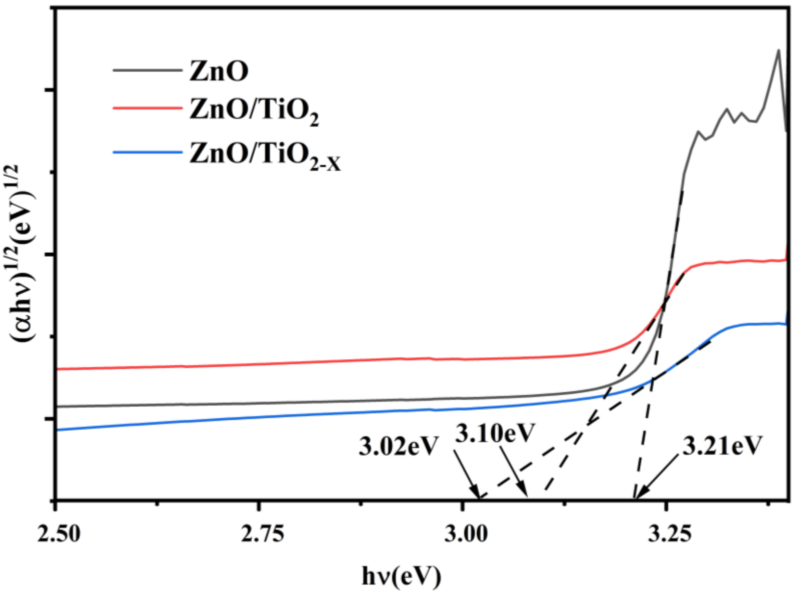

Figure S4. The plot of $(\alpha h\nu)^{1/2}$ versus the energy of light (hν).

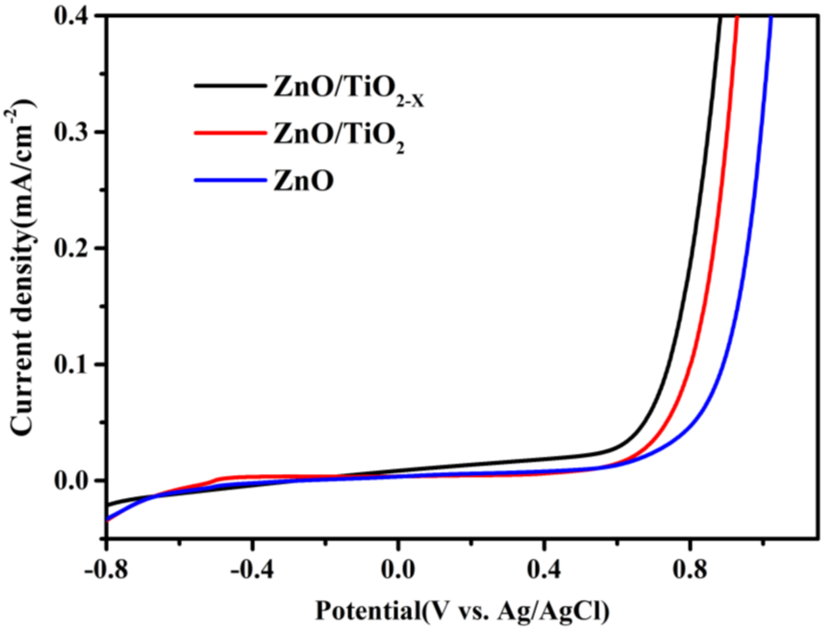

Figure S5. Linear sweep voltammogram under dark conditions.

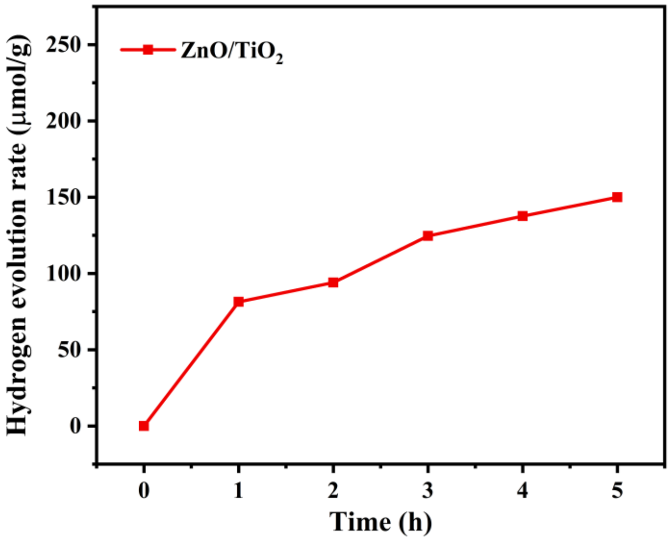

Figure S6. Temporal H_2 evolution evolution of the ZnO/TiO_2.
